# Supplementary material for: Optical Properties of Polyisocyanurate–Polyurethane Aerogels: Study of the Scattering Mechanisms
Source: Nanomaterials (Basel). 2022 Apr 30;12(9):1522. doi: 10.3390/nano12091522 (PMC9100237; doi:10.3390/nano12091522)
Supplement: Supplementary file 1 [file nanomaterials-12-01522-s001.zip › nanomaterials-1689487-supplementary.pdf]

nanomaterials

MDPI

Supplementary Materials

Optical Properties of Polyisocyanurate–Polyurethane Aerogels: Study of the Scattering Mechanisms

Beatriz Merillas [1,*], Judith Martín-de León [1], Fernando Villafañe [2] and Miguel Ángel Rodríguez-Pérez [1,3]

[1] Cellular Materials Laboratory (CellMat), Condensed Matter Physics Department, Faculty of Science, University of Valladolid, Campus Miguel Delibes, 47011 Valladolid, Spain; jmadeleon@fmc.uva.es (J.M.-d.L.); marrod@fmc.uva.es (M.Á.R.-P.)

[2] GIR MIOMeT-IU Cinquima-Química Inorgánica. Faculty of Science, University of Valladolid, Campus Miguel Delibes, 47011 Valladolid, Spain; fernando.villafane@uva.es

[3] BioEcoUVA Research Institute on Bioeconomy, University of Valladolid, 47011 Valladolid, Spain

* Correspondence: b.merillas@fmc.uva.es; Tel.: +34-983423194

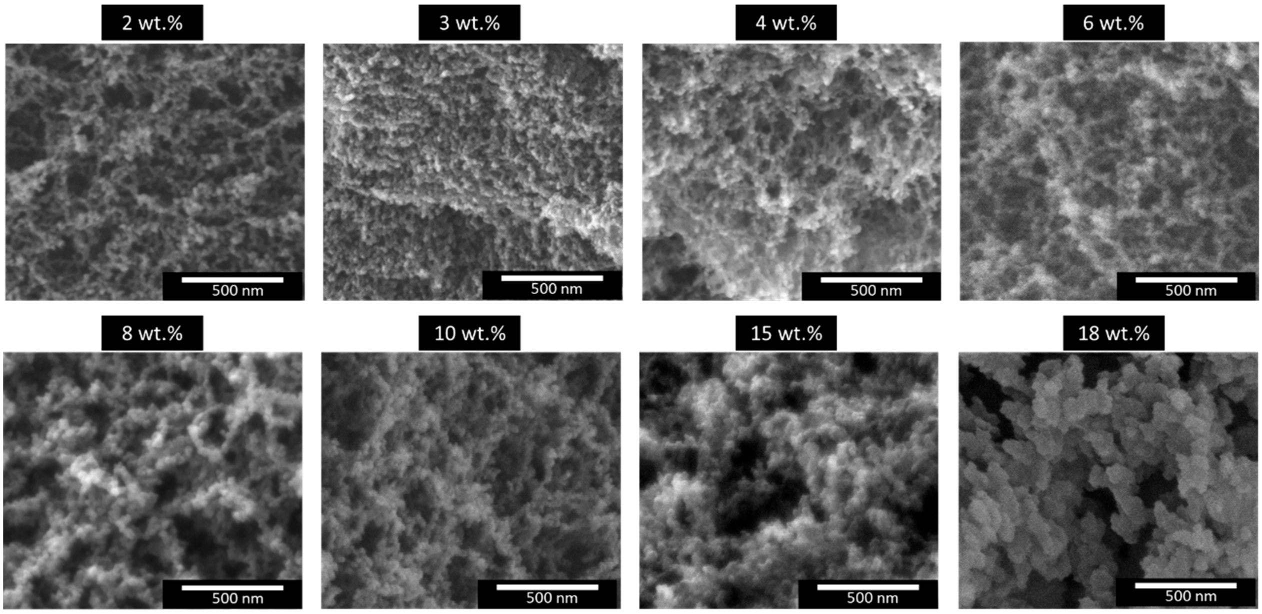

Figure S1. Scanning electron micrographs for all the samples under study at a higher magnification.

Table S1. Parameters obtained by nitrogen sorption measurements: pore volume and specific surface area.

Catalyst amount (wt.%)	V pore (cm^3/g)	S (BET) m^2/g
2	6.25	294.11
3	5.19	288.15
4	5.63	242.09
6	6.24	274.12
8	6.79	197.10
10	7.88	224.28
15	9.04	145.21
18	8.97	49.70

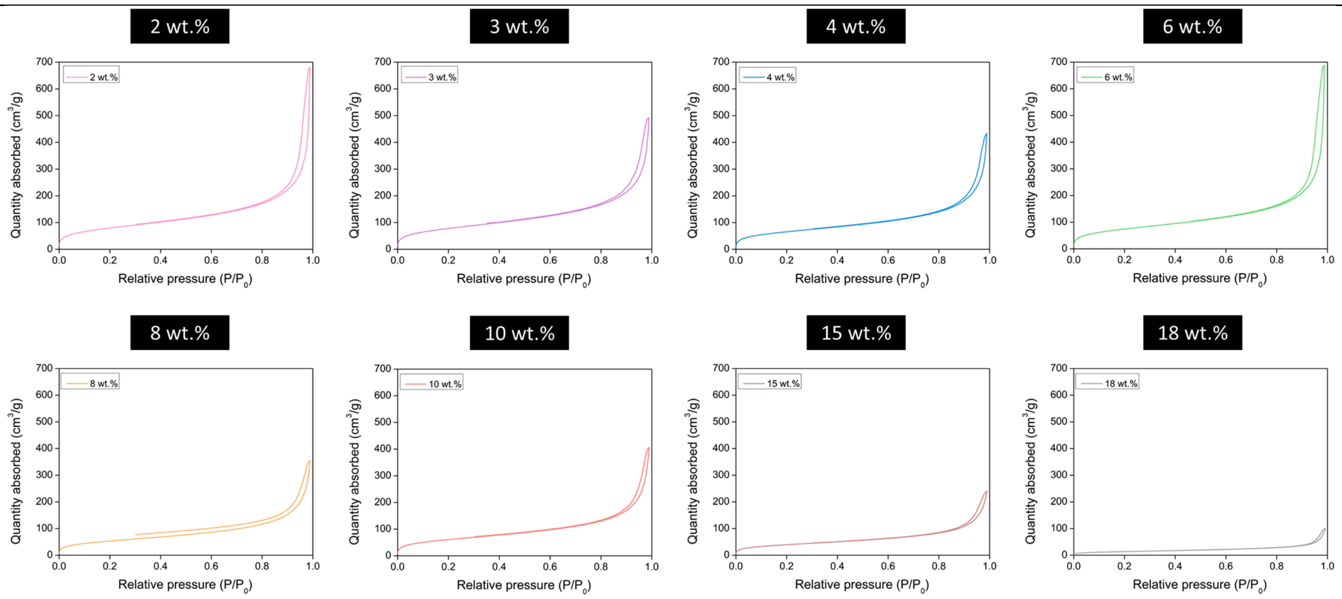

Figure S2. Nitrogen adsorption-desorption curves for all the PUR-PIR aerogels.

Table S2. Adjusted R^2 values for the fitting of -ln(T) as a function of thickness for all the laser wavelengths and all samples.

		R^2	
**wt. %**	**450 nm**	**532 nm**	**650 nm**
2	0.993	0.998	0.993
3	0.989	0.993	0.994
4	0.991	0.996	0.997
6	0.978	0.997	0.963
8	0.957	0.989	0.989
10	0.936	0.972	0.976
15	0.919	0.981	0.992
18	0.904	0.785	0.985

Table S3. Numerical values for the transmittance at three different laser wavelengths for samples of 1 mm thickness.

wt. %	450 nm	532 nm	650 nm
2	0.513	0.709	0.835
3	0.489	0.698	0.774
4	0.379	0.591	0.686
6	0.221	0.474	0.503
8	0.140	0.342	0.428
10	0.139	0.338	0.427
15	0.039	0.087	0.070
18	0.014	0.007	0.033

Table S4. Refraction index for the aerogel samples calculated by the Clausius-Mosotti equation.

wt. %	Refraction index
2	1.047
3	1.062
4	1.052
6	1.052
8	1.043
10	1.052
15	1.039
18	1.038

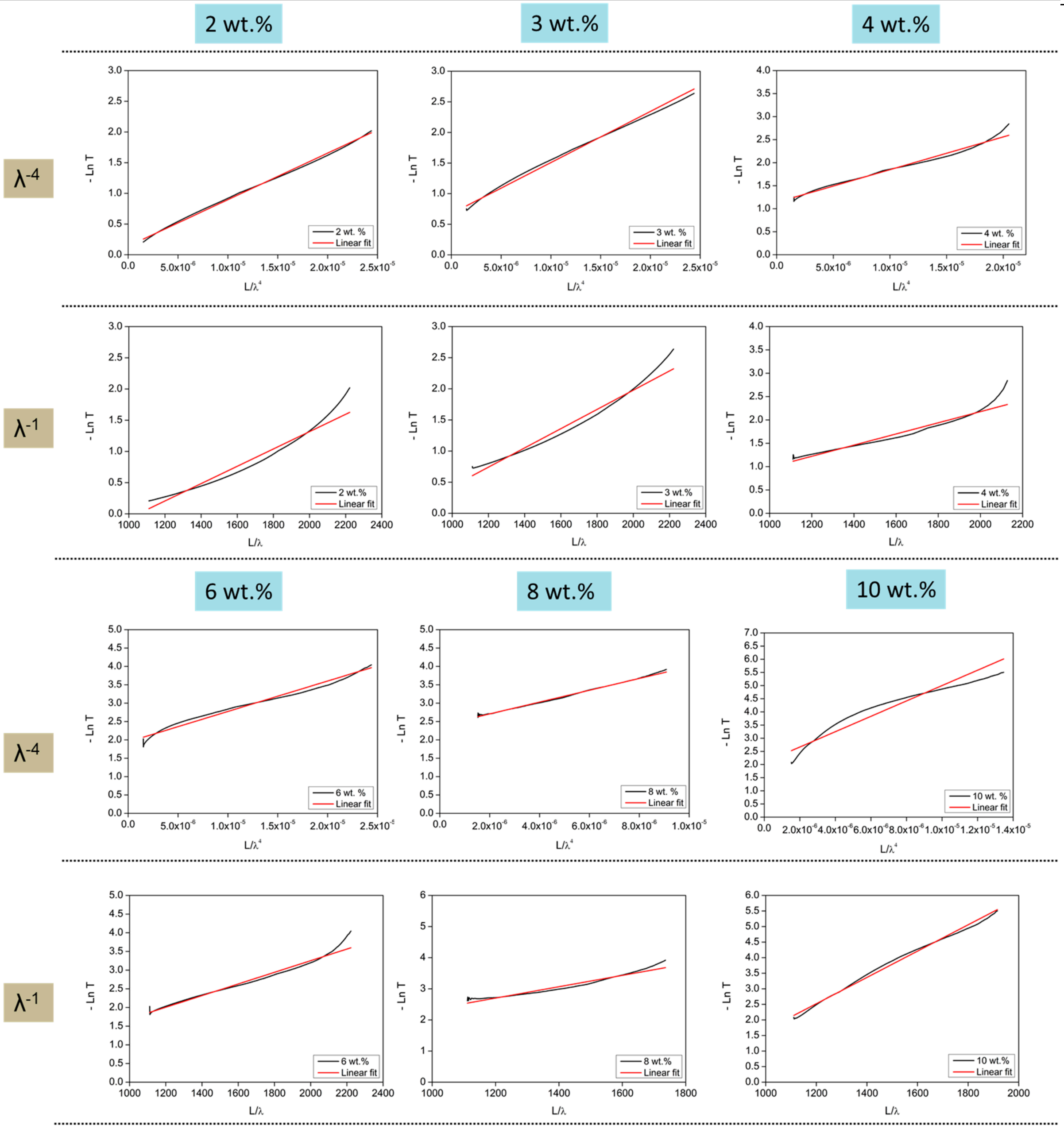

Figure S3. -ln(T) as a function of L/λ⁴ or L/ λ for all the aerogel samples and values of the adjusted R^2 for the linear fitting.

Table S5. Adjusted R^2 values for the fitting of –ln(T) as a function of L/λ⁴ or L/ λ.

	λ^{-4}	λ^{-1}
wt. %	R^2	R^2
2	0.999	0.968
3	0.997	0.980
4	0.987	0.940
6	0.992	0.964
8	0.996	0.968
10	0.977	0.991
